# Myogenesis of *Siboglinum fiordicum* sheds light on body regionalisation in beard worms (Siboglinidae, Annelida)

**DOI:** 10.1186/s12983-021-00426-9

**Published:** 2021-09-16

**Authors:** Nadezhda Rimskaya-Korsakova, Nadezda Karaseva, Timofei Pimenov, Hans Tore Rapp, Eve Southward, Elena Temereva, Katrine Worsaae

**Affiliations:** 1 Department of Invertebrate Zoology, Faculty of Biology, Lomonosov Moscow State University, Moscow, Russia; 2 Centre for Geobiology and Department of Biology, University of Bergen, Bergen, Norway; 3 Marine Biological Association of the U.K., Citadel Hill, Plymouth, PL1 2PB UK; 4 Faculty Biology and Biotechnology, National Research University Higher School of Economics, Moscow, Russia; 5 Marine Biological Section, Department of Biology, University of Copenhagen, Copenhagen, Denmark

**Keywords:** Confocal laser scanning microscopy, F-actin, Transmission electron microscopy, Septa, Ultrastructure, Frenulate pogonophorans, Tagmata, Opisthosoma

## Abstract

**Background:**

Many annelids, including well-studied species such as *Platynereis*, show similar structured segments along their body axis (homonomous segmentation). However, numerous annelid species diverge from this pattern and exhibit specialised segments or body regions (heteronomous segmentation). Recent phylogenomic studies and paleontological findings suggest that a heteronomous body architecture may represent an ancestral condition in Annelida. To better understand the segmentation within heteronomous species we describe the myogenesis and mesodermal delineation of segments in *Siboglinum fiordicum* during development.

**Results:**

Employing confocal and transmission electron microscopy we show that the somatic longitudinal musculature consists of four separate strands, among which ventrolateral one is the most prominent and is proposed to drive the search movements of the head of the late metatrochophore. The somatic circular musculature lies inside the longitudinal musculature and is predominantly developed at the anterior end of the competent larva to support the burrowing behaviour. Our application of transmission electron microscopy allows us to describe the developmental order of the non-muscular septa. The first septum to form is supported by thick bundles of longitudinal muscles and separates the body into an anterior and a posterior region. The second group of septa to develop further divides the posterior body region (opisthosoma) and is supported by developing circular muscles. At the late larval stage, a septum reinforced by circular muscles divides the anterior body region into a forepart and a trunk segment. The remaining septa and their circular muscles form one by one at the very posterior end of the opisthosoma.

**Conclusions:**

The heteronomous *Siboglinum* lacks the strict anterior to posterior sequence of segment formation as it is found in the most studied annelid species. Instead, the first septum divides the body into two body regions before segments are laid down in first the posterior opisthosoma and then in the anterior body, respectively. Similar patterns of segment formation are described for the heteronomous chaetopterid *Chaetopterus variopedatus* and serpulid *Hydroides elegans* and may represent an adaptation of these annelids to the settlement and transition to the sedentarian-tubiculous mode of life.

**Supplementary Information:**

The online version contains supplementary material available at 10.1186/s12983-021-00426-9.

## Background

The body plan of annelids is in textbooks often presented as a regular series of segments, similar in structure and function. Such a homonomous (similar) organization is demonstrated in the well-studied Errantian model annelid *Platynereis dumerilii* [1, 2]. However, many annelids (as well as arthropods) have heteronomous (irregular) segmentation, where groups of segments are specialized to perform certain functions, for example, reproduction, mating, feeding, gas exchange, tube construction, etc. [3, 4]. Such a "division of labor", manifested in the functional specialization of different parts of the body, contributed to the successful diversification of annelids. Differences along the body axis are expressed in the arrangement of chaetae, parapodia (lateral outgrowths of the body), coelomic cavities and coelomoducts, musculature, and the nervous system [5–7]. These differences are already evident in several early branching lineages of annelids, such as Oweniidae, Mageloniidae, Psammodrilidae, and Chaetopteridae [8–14]. The recently discovered Cambrian endobentic magelonid-like annelid *Dannychaetae* also possess a heteronomously segmented body [15]. The phylogenetic position and old origin of these annelid taxa urge for new comparative analyses and ancestral reconstruction of segmental organisation in annelids, which might not have been homonomous as previously suggested [16–19]. Developmental studies of heteronomous representatives are therefore essential for identifying possible common formation schemes across annelids and reconstruct the evolution of segmental organization in Annelida.

The iconic family of deep-sea gutless worms, Siboglinidae, has a heteronomous body architecture. Siboglinids are found in reducing habitats and includes Vestimentifera (hydrothermal vents and hydrocarbon seeps), *Sclerolinum* (sediments, and decaying organic matter), *Osedax* (from the decaying vertebrate bones, and sediment beneath), and Frenulata (from seeps and sediment enriched with sulphides) [20–24]. Siboglinidae are nested within Sedentaria in Pleistoannelida [8, 9]. Within Siboglinidae, the frenulate pogonophorans constitute the sister clade to the remaining siboglinids [25]. The frenulates are characterised by the presence of a small cephalic lobe, a forepart bearing frenulum and tentacle(s), a highly elongated trunk (encompassing maturing gametes and endosymbiotic bacteria), and an opisthosome, with chaetae-bearing short segments. However, for a long time frenulates were considered as deuterostomes, consisting of three body parts [26, 27], and only in the nineties did molecular based analyses finally confirm their affinity to Annelida (e.g., [28]). Detailed comparative analyses of the body plan of frenulate pogonophorans in the light of their annelid affinity have not yet been carried out.

Current knowledge on the anatomy of siboglinid larvae is mainly based on ultrastructural studies of vestimentiferans [29–33], and immunolabeling and confocal scanning microscopy on larva-like male of *Osedax* [34]. Only few histological [35–37] and ultrastructural [38] data on frenulate larvae exist and their full anatomy needs to be reconstructed with advanced microscopy techniques and considering their current position within Annelida.

Frenulate pogonophorans, forming a sister clade to the remaining siboglinids, provide an excellent model for studying heteronomous development in this group. We have focused on muscle development, which as mesodermal tissue set the segment borders prior to the appearance of ectodermal borders [39–43]. The myogenesis of *Siboglinum fiordicum* is mapped and reconstructed with F-actin (phalloidin) staining and confocal laser scanning microscopy and ultrastructural details investigated employing transmission electron microscopy. The structure and development of musculature and septa shed light on the regionalization of the body and temporal formation of segments along the anterior–posterior axis. Videorecording of live animals provides information on larval motility patterns that in combination with the morphological data allow us to hypothesize on the functional adaptation to the larval lifestyle of the of frenulate pogonophorans, and particularly *Siboglinum fiordicum*.

## Results

**Fig. 1:**
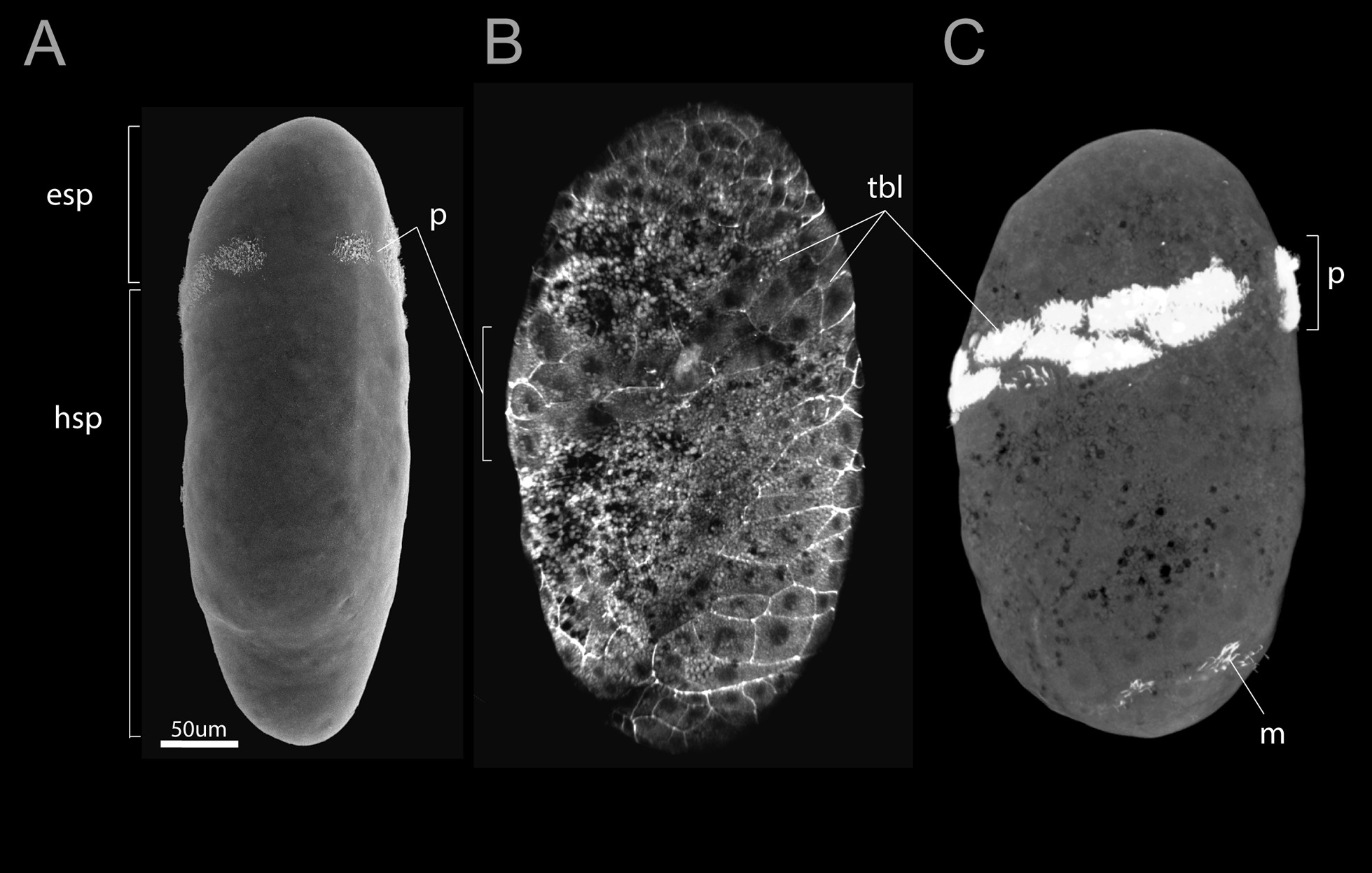
Trochophores of *Siboglinum fiordicum*. **A** An early trochophore with prototroch (SEM). **B**, **C** Late trochophore with the prototroch and irregular mesotroch, CLSM of the staining with phalloidin (**B**) and anti-α-tubulin (**C**). esp—episphere, hsp—hyposphere, m—mesotroch, p—prototroch, tbl—trochoblasts

**Fig. 2:**
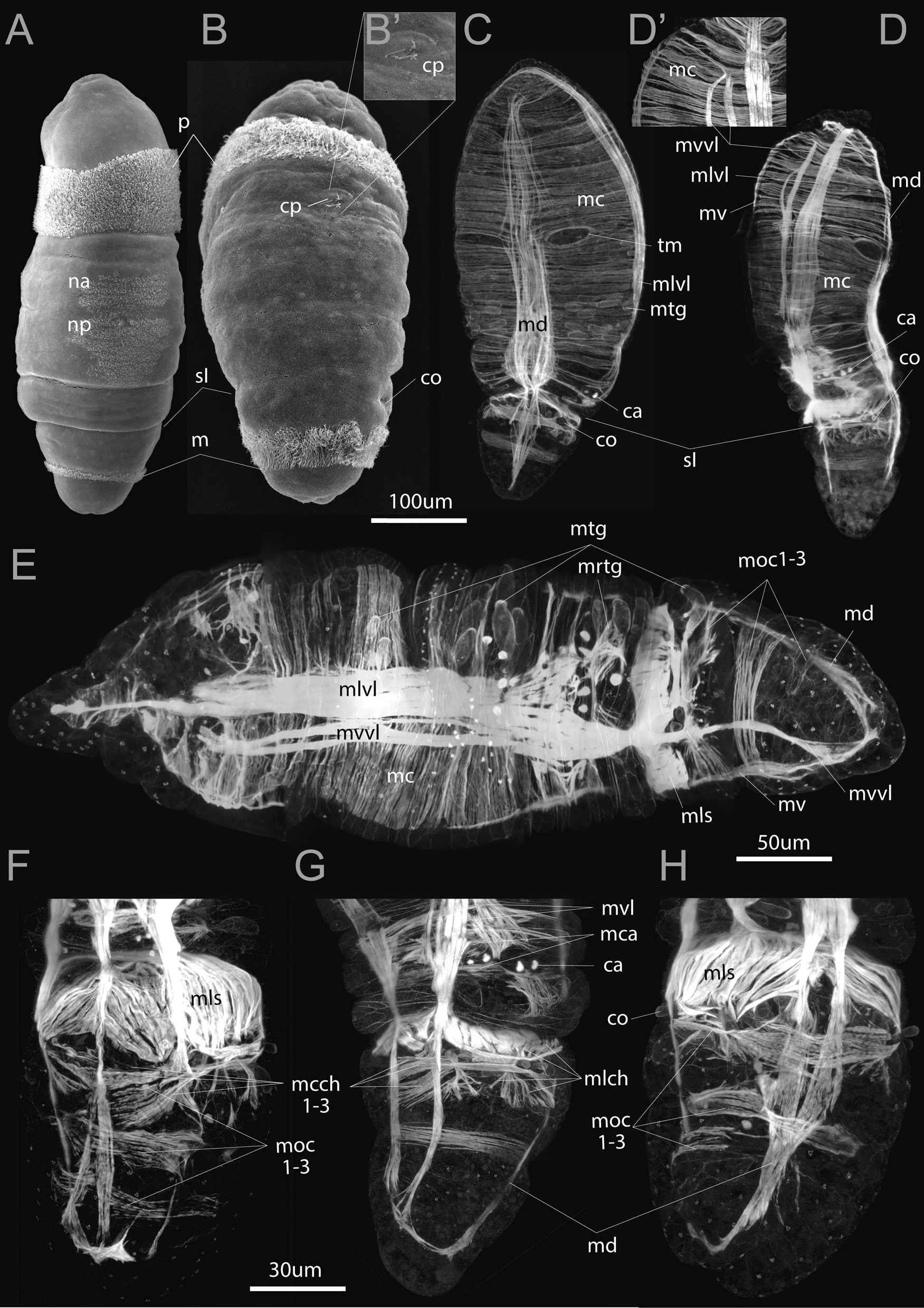
Early metatrochophores of *Siboglinum fiordicum,* by SEM (**A**, **B**) and phalloidin staining and CLSM (**C**–**H**). **A**, **B** Ventral and dorsal view of the larva in that the first septum (*sI*) internally divides body into two tagmata: common anterior segment and common opisthosomal segment. Dorsal side distinguished by the presence of the ciliated spot (*cp*) (**B**). **C** Dorsal view of the larva, distinguished by the presence of the single dorsal longitudinal muscle strand (*md*) and asymmetric tentacular muscles (*tm*). **D**, **E **Left lateral view distinguished by the presence of the ventrolateral longitudinal muscle strands (*mvvl, mlvl*) and layer of circular muscles in the anterior part of the body. Circular muscles encircle the body and overlap on the ventral (**D’**) side of the body. **F**–**H** Ventral, lateral, dorsal views of the opisthosoma bearing three first circular bands (*moc1-3*). ca—annular chaetae, co—opisthosomal chaetae, cp—dorsal ciliated spot, m—mesotroch, mc—circular musculature, mca—muscles of annular chaetae, mcch1-3—circular muscles of the chaetal apparatus, md—dorsal longitudinal muscle strand, mlch—longitudinal muscles of the chaetal apparatus, mls—longitudinal muscle of the first septum, mlvl—lateral component of the ventrolateral longitudinal muscle strand, moc1-3—circular muscles in the opisthosoma, mrtg—radial muscles interconnecting mtg and longitudinal muscle strands, mtg—muscles of the tubiparous glands, mv—ventral longitudinal muscle strand, mvvl—ventral component of the ventrolateral longitudinal muscle strand, na—anterior neurotroch, np—posterior neurotroch, p—prototroch, sI—place of the first septum, tm—muscle bundles of the tentacular anlage

**Fig. 3:**
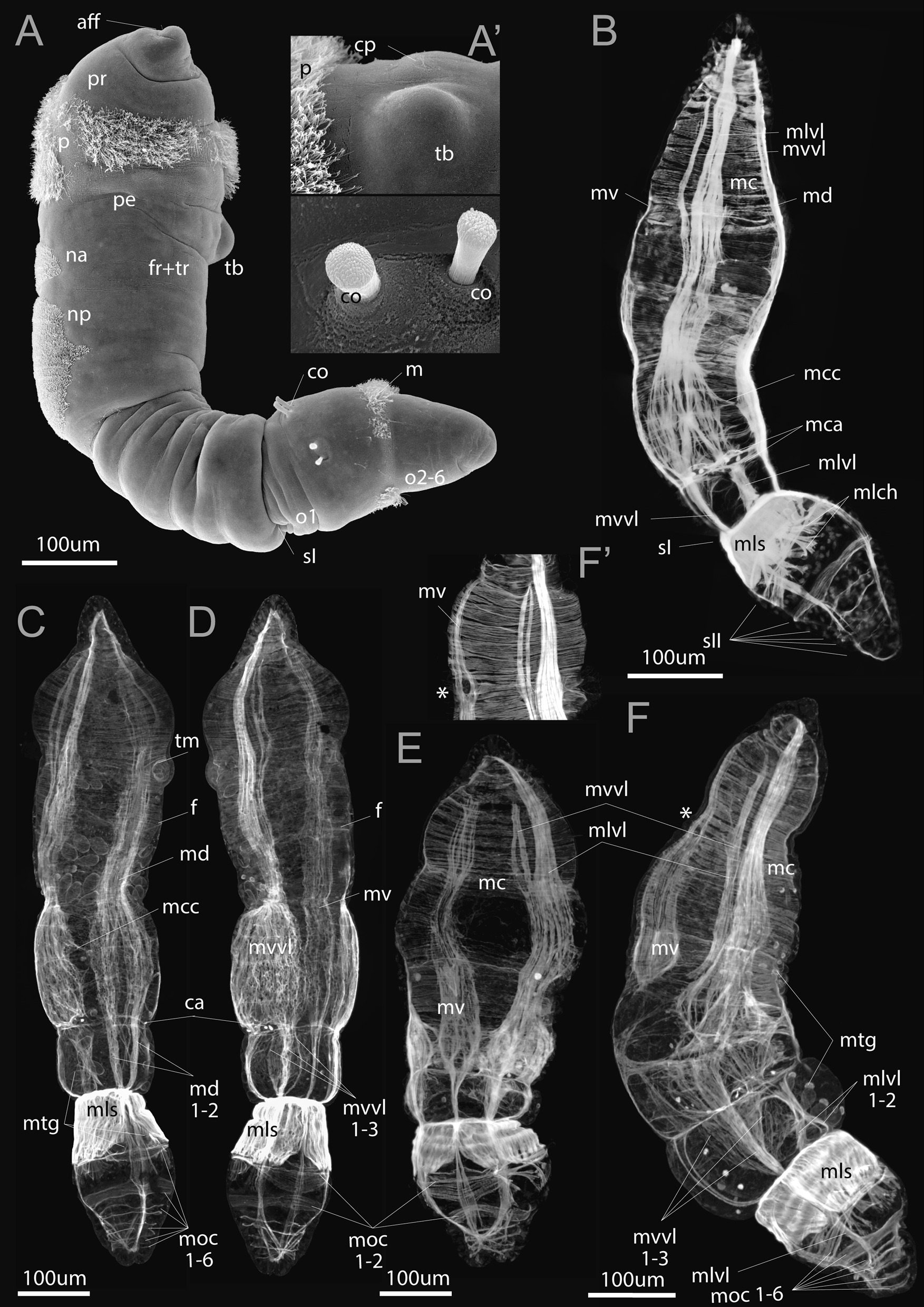
Late metatrochophores of *Siboglinum fiordicum,* by SEM (**A**) and phalloidin staining and CLSM (**B**-**F**). **A** Lateral view of the larva with the visible first septum dividing common anterior segment (*fr + tr*) from the segmented opisthosoma (*o1, o2-6*), it has opisthosomal chaetae (*co*) and bud of future tentacle (*tb*) which are also shown in (**A’**). **B–D** Lateral, dorsal and ventral views of the late metatrochophores with well-developed longitudinal and circular musculature. At this stage, the muscles of the first septa remarkably increase, six circular bands marking position of the opisthosomal septa are formed, the musculature of the first opisthosomal chaetae is formed. **E**, **F** Ventral and lateral view of the larvae showing muscle contractions during movement. Late metatrochophores with holes in the ventral strand (**F’**) which is a reminiscence of the mouth which was open and functional in the ancestors (shown by*). aff—frontal fold, ca—annular chaetae, co—opisthosomal chaetae, cp—dorsal ciliated spot, f—frenulum, fr + tr—common anterior segment, m—mesotroch, mc—circular musculature, mca—muscles of annular chaetae, mcc—lateral circular muscle fibers interconnecting the longitudinal strands, md—dorsal longitudinal muscle strand, md1-2—two components of md in the postannular trunk segment, mlch—longitudinal muscles of the chaetal apparatus, mls—longitudinal muscle of the first septum, mlvl—lateral component of the ventrolateral longitudinal muscle strand, mlvl1-2—two components of mlvl in the postannular trunk segment, moc1-6—circular muscles in the opisthosoma, mtg—muscles of the tubiparous glands, mv—ventral longitudinal muscle strand, mvvl—ventral component of the ventrolateral longitudinal muscle strand, mvvl1-3—three components of mvvl in the postannular trunk segment, na—anterior neurotroch, np—posterior neurotroch, p—prototroch, pe—peristomium, pr—prostomium, sI—the first septum in the larval ontogenesis, sII—the second septum in order of formation, tb—tentacular anlage, tm—muscle bundles of the tb

**Fig. 4:**
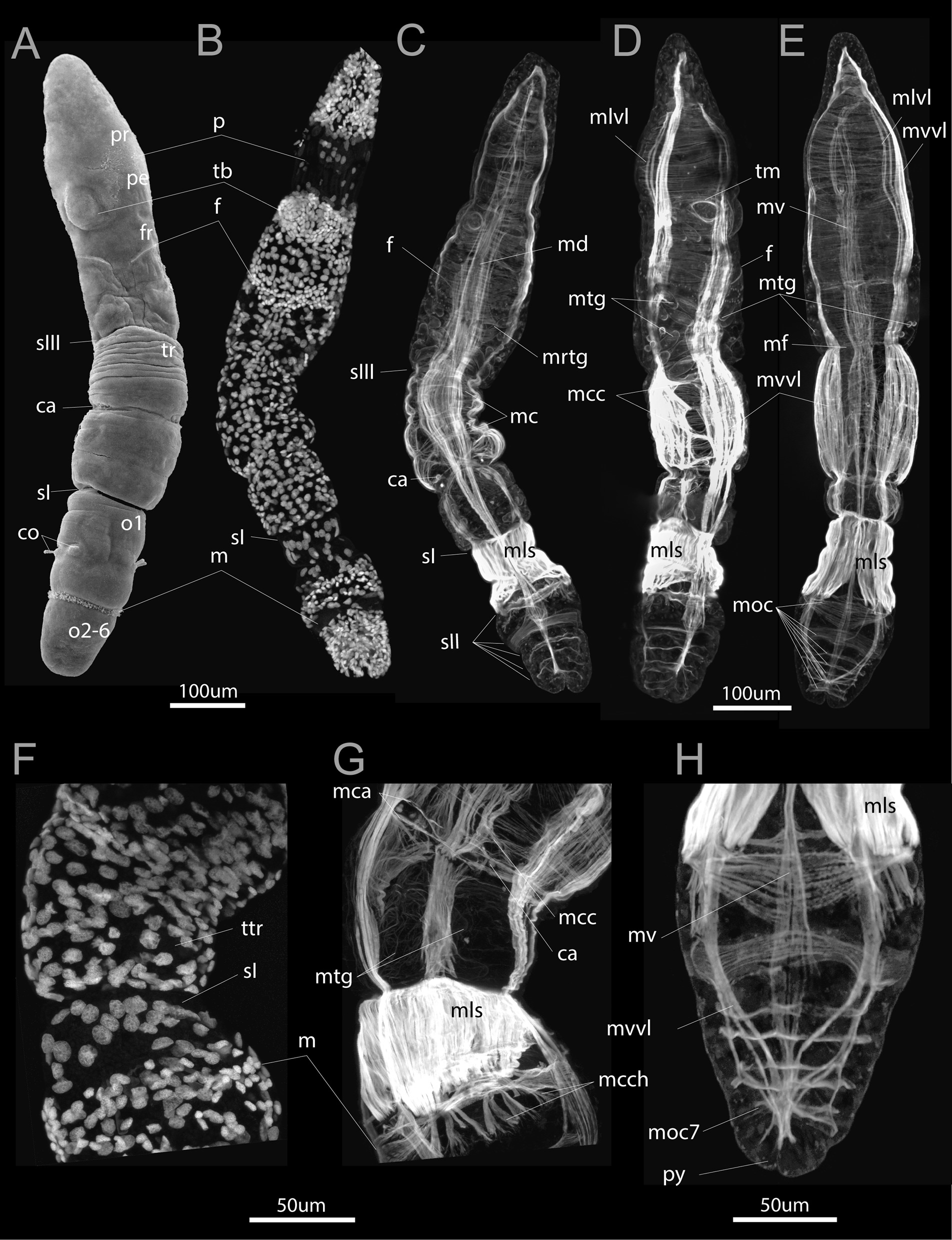
Competent larva of *Siboglinum fiordicum,* by SEM (**A**) and phalloidin and DAPI staining visualized by CLSM (**B–H**). **A** Dorsal view of the competent larva having the prostomium (*pr*), peristomium (*pe*), forepart segment (*fr*), trunk segment (*tr*), ca 6 opisthosomal segments (*o1, o2-6*). **C** DAPI staining marks the accumulation of cells in the region of the tentacle rudiment (tb), frenulum (*f*), and vice versa, the rare location of large cells, such as trochoblasts of the prototroch (*p*) and mesotroch (*m*), and myoepithelial cells that form the muscles of the first septum (*sI*). **C–E** Dorsal, lateral and ventral views of the competent larvae with the circular musculature in the anterior end and almost complete layers of the prominent longitudinal musculature in the posterior end of larva. Note in (**C**, **D**) the periglandular musculature (*mtg*) which are equipped with the radial fibers extending from the main longitudinal strand (*mrtg*). **F**, **G** DAPI and phalloidin stainings the postannular area of the trunk segment (*ttr*), which will encompass the trophosome in juveniles. **H** Ventral view of the opistosoma, the seventh circular muscle added (*moc7*) at the posteriormost end, pygidium (*py*) is hardly distinguished. ca—annular chaetae, co—opisthosomal chaetae, f—frenulum, fr—forepart, m—mesotroch, mc—circular musculature, mca—muscles of annular chaetae, mcc—lateral circular muscle fibers interconnecting the longitudinal strands, mcch—circular muscles of the chaetal apparatus, md—dorsal longitudinal muscle strand, mf—anterior circular muscle of the sIII, mls—longitudinal muscle of the first septum, mlvl—lateral component of the ventrolateral longitudinal muscle strand, moc—circular muscles in the opisthosoma, mrtg—radial muscles interconnecting mtg and longitudinal muscle strands, mtg—muscles of the tubiparous glands, mv—ventral longitudinal muscle strand, mvvl—ventral component of the ventrolateral longitudinal muscle strand, o1-o2—the first-sixth segments of the opisthosoma, p—prototroch, pe—peristomium, pr—prostomium, py—pygidium, sI—the first septum in the larval ontogenesis, sII—the second septum in order of formation, sIII—the third septum in order of formation, tb—tentacular anlage, tm—muscle bundles of the tb, tr—trunk segment

**Fig. 5:**
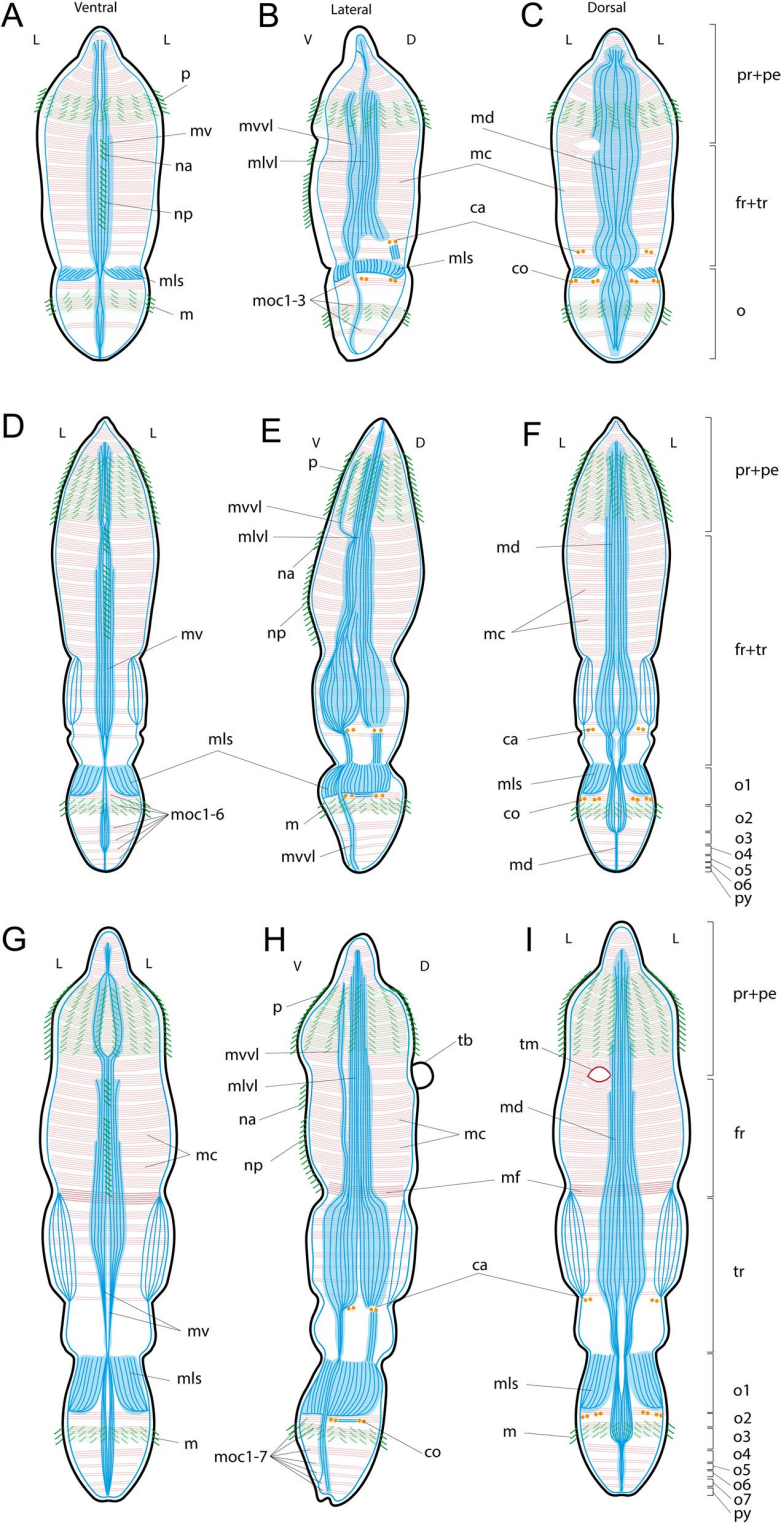
Schematic drawings of the myognesis in *Siboglinum fiordicum* larvae. **A**–**C** Early metatrochophore, the first septum divides body into two tagmata: common anterior segment (*fr + tr*) and common opisthosomal segment (*o*), **D–F** Late metatrochophore, division of the opisthosoma into segments, **G–I** competent larva, division of the common anterior segment into forepart (*fr*) and trunk (*tr*) and new posterior segments appear at the end of the opisthosoma. Longitudinal muscles are shown in blue, circular muscles are in red, cilia—in green, chaetae—in orange. L, V, D serve for lateral, ventral and dorsal body orientations. ca—annular chaetae, co—opisthosomal chaetae, fr—forepart segment, m—mesotroch, mc—circular musculature, md—dorsal longitudinal muscle strand, mls—longitudinal muscle of the first septum, mlvl—lateral component of the ventrolateral longitudinal muscle strand, moc1-7—the first-seventh circular muscles in the opisthosoma, mv— ventral longitudinal muscle strand, mvvl—ventral component of the ventrolateral longitudinal muscle strand, na—anterior neurotroch, np—posterior neurotroch, o—common segment of the opisthosoma, o1-o7—the first-sixth segments of the opisthosoma, p—prototroch, pe—peristomium, pr—prostomium, py—pygidium, tb—tentacular anlage, tm—circular muscles of the tb, tr—trunk segment

To study the development of muscles in *Siboglinum fiordicum*, we reconstructed the muscular architecture and anatomy in four successive stages: trochophore, early metatrochophore, late metatrochophore, and competent larva (Figs. 1A, 2A, 3A, 4A, 5A–I).

## Trochophore

The size of the trochophores is ca 300–400 um. The widest part of the larva is in the middle of the body (Fig. 1A–C). The trochophore larva has a small convex episphere (*esp*) (without an apical tuft of cilia) and an unusually long hyposphere (*hsp*). The earliest trochophore stage has a thin, irregular prototroch (*p*). In the late trochophore, an irregular strip of mesotroch (*m*) appears (Fig. 1C), and a wide prototroch is formed by large multicilated trochoblast cells (*tbl*) arranged in 2–3 rows (Fig. 1B–C). No muscle fiber is revealed at this stage. The characteristic movement of the trochophore larvae can be seen in the videofile, Additional file 1. Trochophores float due to the beating of their cilia, the body does not bend. They actively swim out of the maternal tube and can spin and swim on the bottom of the dishes.

*Mesotroch* We define the posterior ciliary band in the frenulate larva as a mesotroch, which is the large and complete circular ciliary bands that eventually come to lie within the segmented body [44]. This fact makes mesotroch different from the metatroch and telotroch. Also, this band should not be termed as a paratroch, which forms after the formation of the telotroch [42, 45].

## Early metatrochophore

**Fig. 6:**
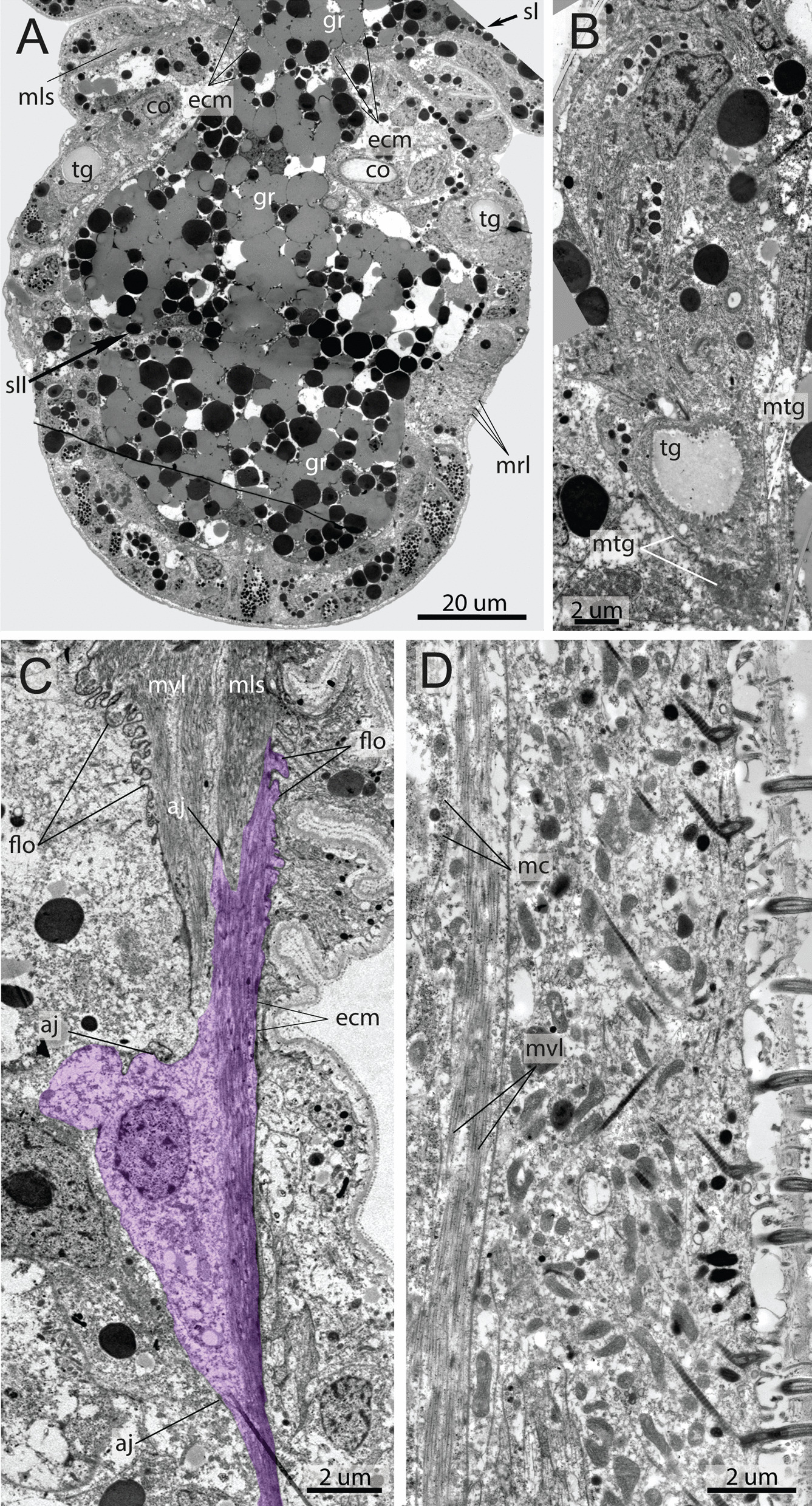
Ultrastructure of somatic musculature, the parafrontal sections of the early metatrochophore (**A**, **C**, **D**) and parasagittal sections of late metatrochophore (**B**) of *Siboglinum fiordicum*. **A** The opisthosomal tagma with the incomplete first septum and with the growing second septum. Through the lumen in the first septum the gut rudiments passes. **B** Tubiparous glands secreting the tube material, note the cup-shaped microvilli facing he lumen of the gland. Thin layer of the muscle surrounding the gland, especially around the developing glandular duct, which supposedly pushes the tube secrete outside when muscles contract. **C** Left ventrolateral longitudinal muscle *(mvl)* strand extending from the trunk segment to the first segment of opisthosoma, and septal longitudinal muscle *(mls)* bands extending from the septum I to the opisthosome. Coelom of opisthosoma lined with the large myoepithelial cells (marked in purple). **D** Circular muscles (*mc*) lie inner to the longitudinal muscles, at the prototroch level. aj—adherens junction, co—opisthosomal chaetae, ecm—extracellular matrix, gr—gut rudiment, flo—finger-like outgrowths, mc—circular muscles, mls—longitudinal muscles of the first septum, mrl—rootlets of the mesotroch cilia, mtg—musculature of the tubiparous gland, mvl—ventrolateral longitudinal muscle strand, sI—the first septum, sII—the second septum, tg—pouch of the tubiparous gland

The early metatrochophore is up to 500 um long (Fig. 2A, B). The body of this stage is divided into joint prostomium + peristomium, a forepart + trunk tagma (which is joint 2nd + 3rd segments) and a opisthosomal tagma (joint opisthosomal segments) (Figs. 5A–C, 6A). The wide prototroch consists of the cilia of similar length (has no opposed beating cilia) and is represented by first short (Fig. 2A), then longer cilia (Fig. 2B). The mesotroch becomes a regular annular stripe (Fig. 2A) with cilia soon extending in length and density (Fig. 2B), and a neurotroch and a dorsal ciliated spot (*cp*) appear (Fig. 2A, B). The neurotroch is a wide ventral ciliary field, consisting of two zones, a small anterior (*na*) and a larger posterior (*np*) (Fig. 2A). At this stage, the chaetae of the annula (*ca*) (middle part of “trunk” or 3rd segment) and the chaetae of the first segment of the opisthosoma (*co*) (i.e., the 4th body segment) are formed in the epidermis (Fig. 2B–H). The chaetae are not visible externally but scanning electron micrographs show depressions overlying the future chaetae of the opisthosoma (Fig. 2B).

*The longitudinal musculature* is formed at the early metatrochophore stage and comprises four separate strands located ventrally (*mv*), dorsally (*md*), and ventro-laterally (*mvl*) (Figs. 2, 5A–C). These four main longitudinal muscle strands extend from the prostomium to the posterior end of the opisthosome.

The dorsal (*md*) and ventral (*mv*) longitudinal strands are unpaired for the most of their length (Fig. 2C, D, F). The ventral strand is the narrowest (Fig. 2D, F; also visible in the later stages Figs. 3D, 4E). The widest strands are the ventro-lateral ones, which have two longitudinal components at all studied larval stages: ventral (*mvvl*) and lateral (*mlvl*) (Fig. 2D, E). The ventral component (*mvvl*) originates at the anterior prototroch and insert at the posteriormost body, while the lateral component (*mlvl*) originates at the anterior prostomium and insert at the first septum (Figs. 2E, 5A–C).

**Fig. 7:**
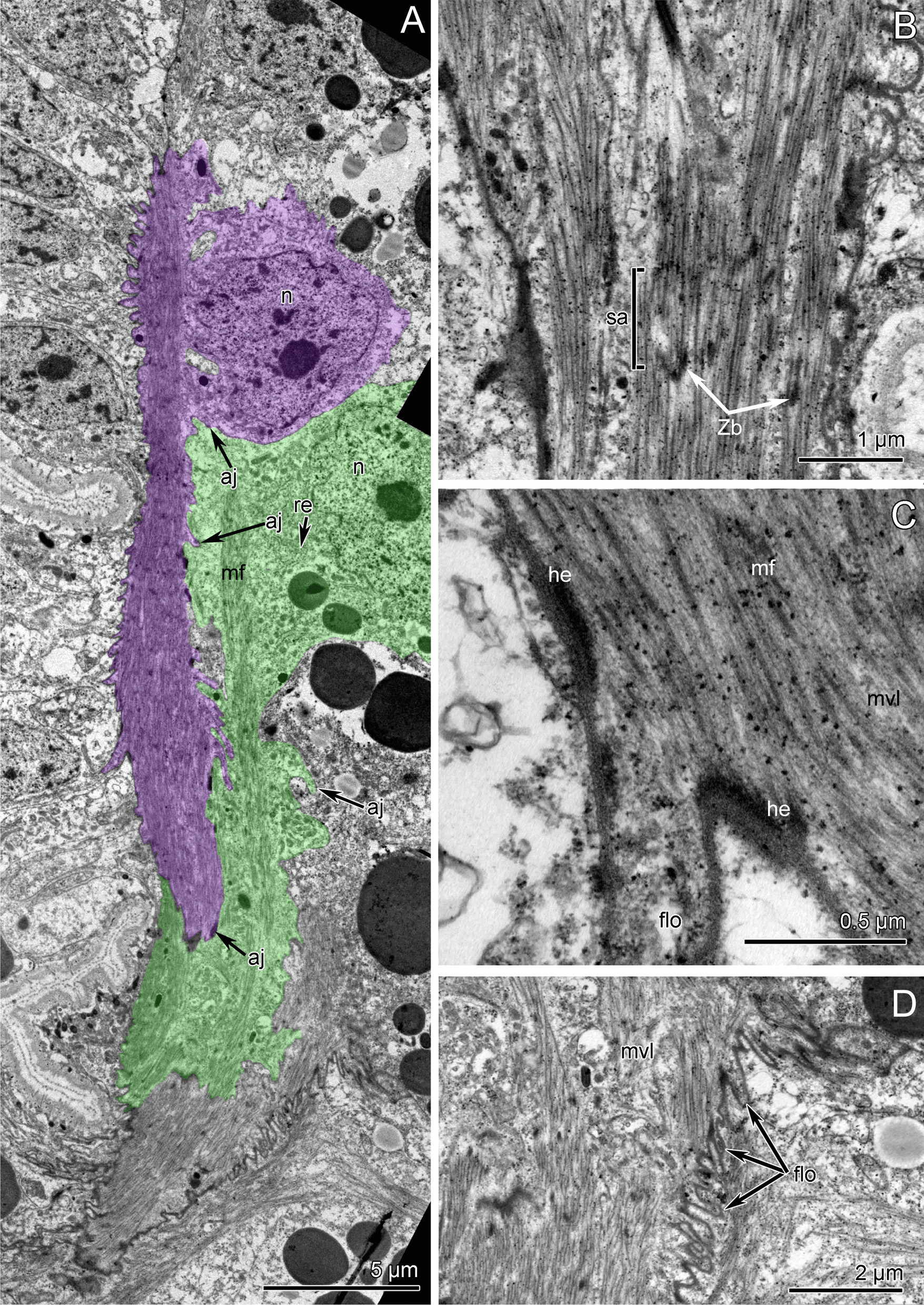
Ultrastructure of the *ventrolateral longitudinal muscle (mvl),* parafrontal sections of the early metatrochophore of *Siboglinum fiordicum*. **A** The myoepithelial cells of the somatic longitudinal musculature form pseudo-multilayer (cells shown in different colors). Highly folded ECM (*flo*) along the contracted right ventrolateral longitudinal muscle (*mvl*). **B** Ultrastructure of the sarcomere of the striated muscles*.* **C** Hemidesmosomes (*he*) of the muscle fibers*.* **D** Site of the highly folded ECM (*flo*) of the septum and basal membrane of the myoepithelial cell: *mvl* from the trunk attaches to the ecm of the septum I. aj—adherens junction flo—finger-like outgrowths, he—hemidesmosomes, mf—longitudinal myofilaments, mls—longitudinal muscles of the first septum, mvl—ventrolateral longitudinal muscle strand, re—rough endoplasmic reticulum, sa—sarcomere, Zb—Z-bodies

The longitudinal muscle fibers are grouped in separate strands, and each strand encompasses certain number of muscle bundles that are unchanged during the development. For example, in the dorsal muscle strand (*md*) there are ca 8–9 bundles (Fig. 2C–E). Due to the presence of the separate strands of the somatic longitudinal musculature, metatrochophores can easily bend their body. So that their ciliary swimming can be accompanied by twisting movements (Additional file 2).

*Circular musculature* (*mc*) as wide bands surrounds the larval body (Fig. 2E). Each circular band includes about eight complete circular muscle bundles. Under the prototroch, the bundles are semi-circular and overlap mid-dorsally and mid-ventrally (Fig. 2C, D’, also visible at later stages: 3F’, 4E). On the dorsal side posterior to the prototroch, the circular muscles form two slightly asymmetrical holes (Fig. 2C). The left hole corresponds to the future unpaired tentacle (*tm*). In the posterior part of the early metatrochophore, the circular muscles are organized in distinctly separate bundles (Fig. 2C, D, F–H).

*The first septum and septal musculature * The first septum (*sI*) divides the body of the early metatrochophore into two parts: a forepart + trunk tagma and a opisthosomal tagma (Figs. 2C, D, 5A–C, 6A). The first septum is formed out of the longitudinal musculature (*mls*, Fig. 2C–H). The longitudinal musculature mainly spread along the lateral sides of the first opisthosomal segment. The muscle bundles stretch between the first septum and the future second septum of the opisthosoma (Fig. 2F–H).

The longitudinal muscles are anchored on a layer of extracellular matrix, which gradually deepens from the body wall inside the body of the larva (Fig. 6A). In the early metatrochophore, the septum is incomplete, and the gut rudiments passes through it (Fig. 6A).

Three anterior circular bands appear in the opisthosoma (*moc1-3*, Figs. 2E, F, H, 5A–C): the first at the level of the first pair of chaetae, the second beneath the mesotroch. The third is located posterior to the second one. These circular bands demarcate the positions of the future second septa (*sII*), which separate anterior opisthosomal segments (4th, 5th, 6th etc.). The early metatrochophore has these three opisthosomal circular muscle bands, but no internal septa yet (Fig. 2E–H), although one of the second septa has started growing (Fig. 6A).

*Musculature of tubiparous glands* is laid down in the early metatrochophore and represented by the muscular sheaths of the sacs and ducts (*mtg*) at all studied stages (Figs. 2C, E, 3F, 4D, E). The glands are located throughout the entire trunk (3rd) segment, and in the first segment of the opisthosoma (Figs. 2E, 3C, 4G). Moreover, the tubiparous glands are associated with radial muscle bundles (*mrtg*), which extend between dorsal and ventrolateral longitudinal muscles strands and distal portion of the gland (Figs. 2E, 4C). Cells of the radial muscles directly abut on the cells of gland lumen (Fig. 6B). The contraction of radial muscles promotes the extrusion of secretion from the lumen of the gland to environment. 

In general, the somatic musculature is formed by myoepithelial cells of the coelomic lining. These are large cells with a prominent apical part and large basal projections (Figs. 6C, 7A). Cells connect via adherence junctions (*aj*) which are located at the border between the apical part of cell and the basal projections and also on the membranes of muscular projections (Figs. 6C, 7A). The apical part of the cell holds a large irregularly shaped nucleus containing small amounts of condensed chromatin and large nucleolus (Figs. 6C, 7A). Cytoplasm around the nucleus is filled with numerous mitochondria of small diameter and electron dense matrix, canals of rough endoplasmic reticulum (*re*), and inclusions of various types. Basal projections of the myoepithelial cells abut on the thin layer of extracellular matrix (*ecm*), which is highly electron dense (Fig. 6D). In some places, which are probably able to stretch extensively (i.e. posterior trunk, Fig. 7A, Additional file 3), the basal membrane of the muscular projections forms numerous finger-like outgrowths (*flo*), which repeat the folds of extracellular matrix and epithelium of body wall (Figs. 6C, 7A). The cytoplasm of muscular projections is filled with numerous myofilaments, which mostly extend longitudinally (Fig. 6D) but can also extend at an angle to the body wall (Figs. 6C, 7B). Myofilaments connect the basal membrane to the cell via electron dense hemidesmosomes (*he*) (Fig. 7C). Myofilaments are organized as in cross-striated musculature: there are sarcomeres (*sa*) and Z-bodies (*Zn*) (Fig. 7B). The cytoplasm of the muscular projections contains mitochondria of small diameter with a few electron dense inclusions (Fig. 7A).

In the somatic longitudinal muscle strands, several myoepithelial cells connect and form a pseudo-multilayer (Fig. 7A). This construction forms because the muscular projections of the cells overlay each other. However, all cells contact the layer of extracellular matrix and, hereby, myoepithelial cells form a monolayer (Fig. 7A). In some places where the myoepithelial cells and their projections contact each other, the cell membranes form numerous finger-like protrusions, which all together work as an interdigitate cell junction (Fig. 7D).

## Late metatrochophore

Late metatrochophores of *S. fiordicum* are noticeably elongated, they reach 700 um long (Fig. 3A). The body consists of prostomium, peristomium, joint forepart + trunk, and up to six segments of opisthosoma (Fig. 5D–F). In the cone-shaped prostomium (*pr*) of the larva, a frontal fold (*aff*) appears on the ventral side (Fig. 5D–F). The peristomium (*pe*), located behind the prototroch, carries a tentacle rudiment (*tb*), which is laid on the dorsal side to the left of the ciliated spot (Fig. 3B). *S. fiordicum* does not have a mouth opening, but a gap in the musculature (Fig. 3F’). The joint tagma, forepart + trunk (= 2nd + 3rd segments), bears anteriorly a neurotroch, comprising an anterior and a posterior zones. Posteriorly, the forepart + trunk tagma is the narrowest part of the body, located behind the middle fold of the body and extending to the first septum which borders the anterior segment of the opisthosoma (Fig. 3A). In the first segment of the opisthosoma, large chaetae are clearly visible and there is a thin mesotroch posterior to the chaetae (Fig. 3A).

*Longitudinal musculature* Changes in the longitudinal musculature include an increase of thickness of each bundle due to the increase in number of muscle fibers that make up the individual bundles. The longitudinal strands gradually spread to form a continuous layer. Also they are interconnected by short lateral circular muscle fibers (*mcc*) (Fig. 3C, also at the later stages: Fig. 4C, D). Late metatrochophores stop swimming by cilia, but they actively bend the body (Additional file 3). Interestingly, the bending movement occurs at the level of the posterior trunk, where the longitudinal muscles are predominantly developed (Figs. 3B, 5D–F), and it is also in this area that we find many finger-like outgrowths of the muscular projections (Figs. 6C, 7A) that allow this part of the trunk to bend and stretch easily (Fig. 3E, F, Additional file 3).

At this developmental stage, the possible place of the mouth opening is visible. Behind the prototroch, the bundles of the ventral longitudinal muscle strand split (Fig. 3F, F’). In the competent larva, the remnants of the hole is still visible (Fig. 4E). Externally, there is no trace of the mouth opening.

The posterior trunk (posterior part of the 3rd segment) lacks the wide longitudinal muscles strands, but there are six dense bundles: a double dorsal one (*md1-2*), and ventral one, while the ventro-lateral muscle strand is represented by two triple (*mvvl1-3*) and two double bundles (*mlvl1-2*, Fig. 3B–D, F).

**Fig. 8:**
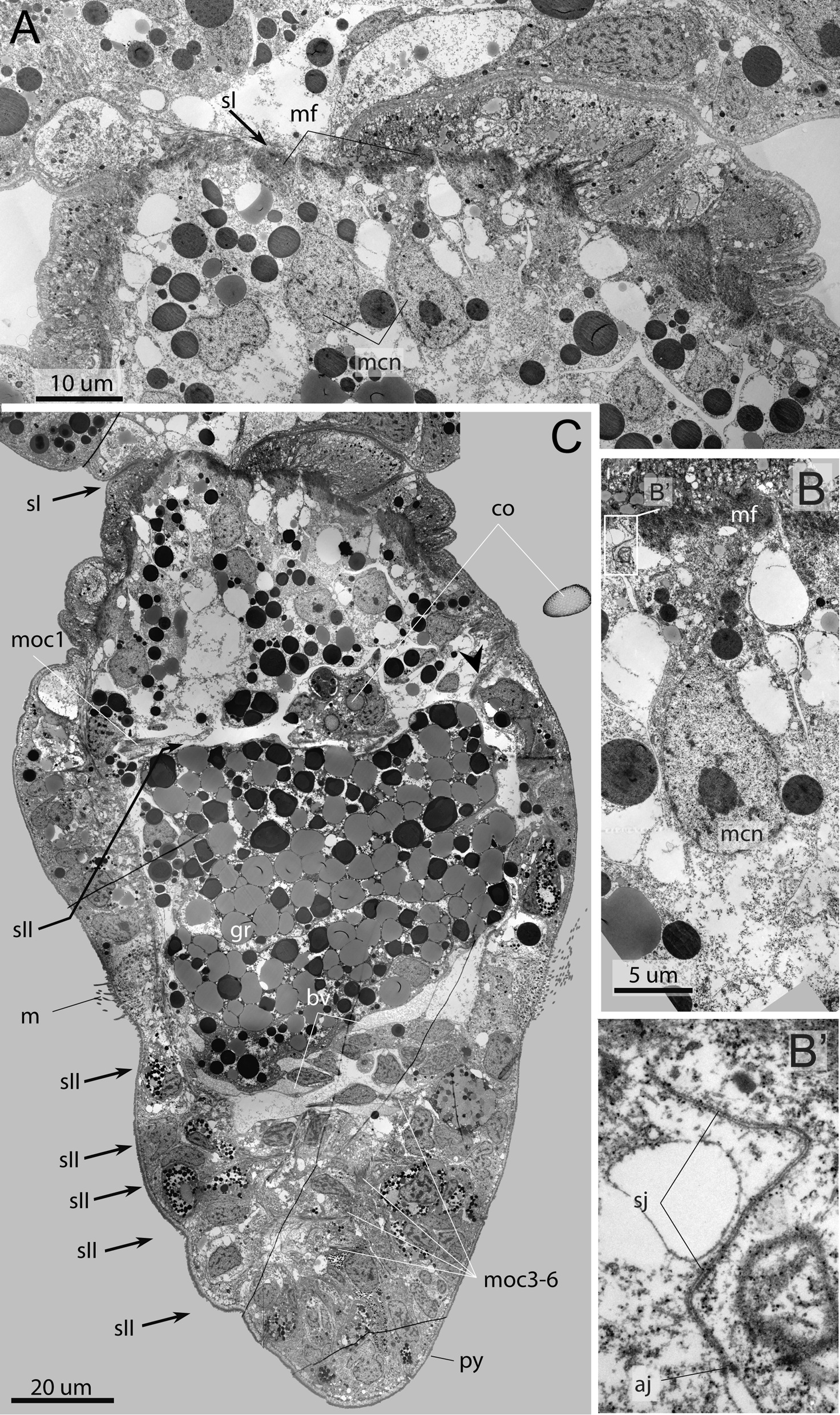
Ultrastructure of the first septum and opisthosomal segments of the late metatrochophore of *Siboglinum fiordicum*, parasagittal sections. **A** The first septum formed by bottleneck-shaped myoepithelial cells. **B** Myoepithelial cell of the first septum bearing the myofilaments. **B**’—cells contacted with adherens junctions (*aj*) and septate junction (*sj*). **C** The sagittal section of the segmented opisthosoma, segments are divided by non-muscular septa, which are the second in order of formation (*sII*), marked by arrowheads. sII at this stage is just thin layers of the ECM (sometimes with blood), they have no muscle fibers, but they contacted to the myoepithelial cells of the body wall (at place of *moc1-6*). aj—adherens junctions, bv—blood vessel, co—opisthosomal chaetae, gr—gut rudiment, m—mesotroch, mcn—large nuclei of the longitudinal myoepithelial cells, mf—myofilaments, moc1-6—the first-sixth circular muscle bundles in the opisthosoma, py—pygidial area, sj—septate junction, sI—the septum that is formed the first in order of formation, dividing trunk and the first opisthosomal segments, sII—the septa that are formed second in order of formation, dividing the opisthosomal segments, simultaneously formed

**Fig. 9:**
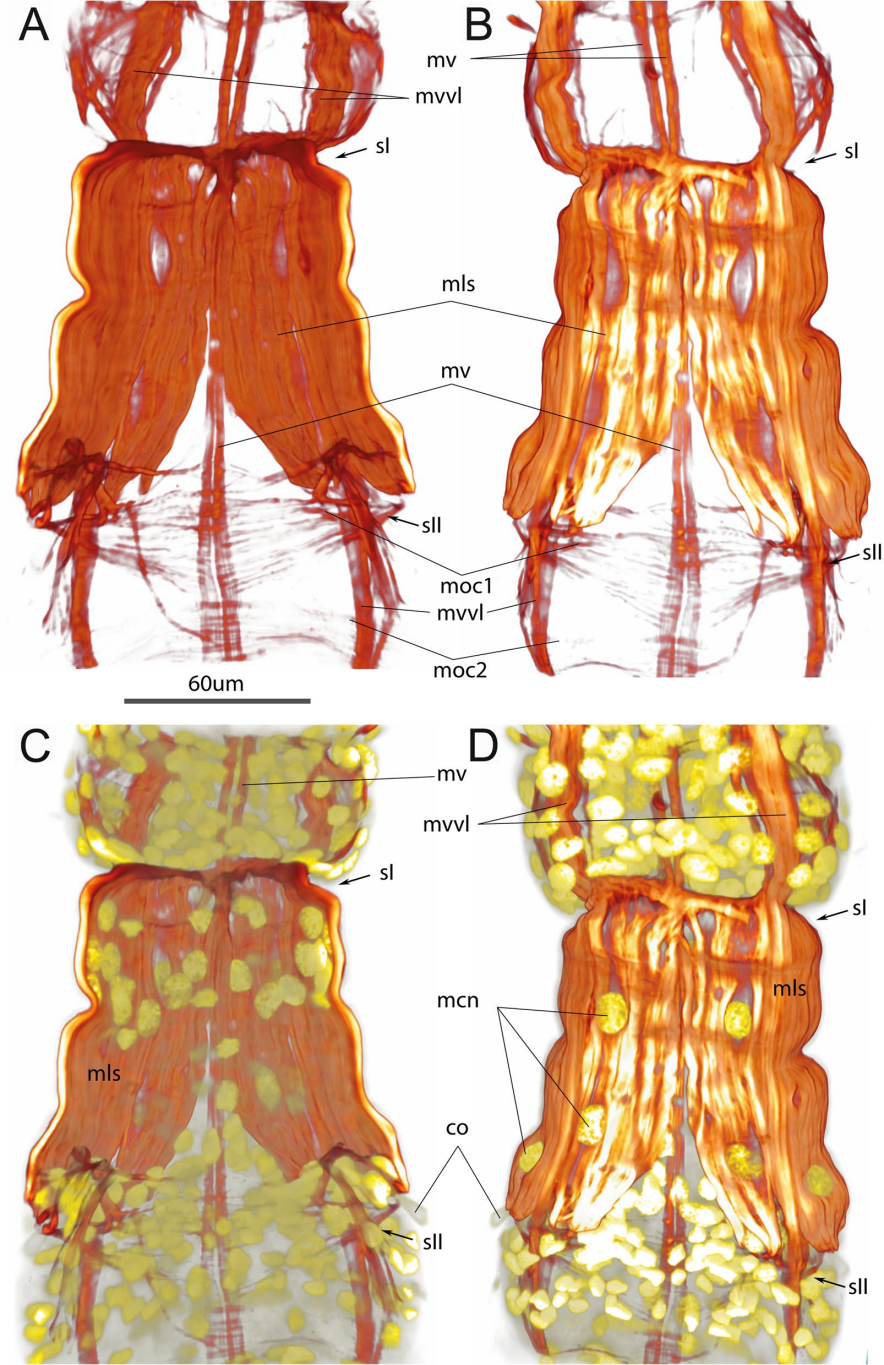
Musculature of the first septum in the competent larva of *Siboglinum fiordicum.* Surface rendering in AMIRA of the longitudinal muscles of the first septum between trunk segment and the first segment of the opisthosoma**.** Musculature is shown in red; nuclei stained with DAPI as well as autofluoresce of the opisthosomal chaetae are shown in yellow. **A**, **C** Ventral view from the inside the body of the larva. **B**, **D** Ventral view from the outside the larva. co—opisthosomal chaetae, mcn—nuclei of large myoepithelial cells of the 1st septum, mls—longitudinal muscles of the first septum, moc1-2—the first and the second circular muscle bundles in opisthosoma, mv—ventral longitudinal strand, mvvl—ventral component of the ventrolateral longitudinal muscle strand, sI—the first septum in the larval ontogenesis, sII—the second septum in order of formation

*The first septum and septal musculature* The formation of the first septum is completed in the late metatrochophore (Fig. 8A, C). The septum completely separates the trunk segment (3rd segment) and the 1st opisthosomal segment (4th segment). The ecm layer occludes and the continuous layer of longitudinal muscles forms (Figs. 5G–I, 8A, C, 9). There are ca 24 pairs of longitudinal bundles building the first septum (Fig. 9A, B). With the larval development the bundles significantly increase in length (compare Fig. 5A–C, G–I). Ca 12 large myoepithelial cells with large nuclei (*mcn*) form this septal muscle (Figs. 8A, B, 9D). The myoepithelial cells of the 1st septum have a large apical part, that extends into the coelom lumen of the 1st opisthosomal segment. Basal parts of the myoepithelial cells contain large nuclei (Fig. 8A, B). These cells are connected not only via adherence junctions, but also by septate junction (Fig. 8B, B’). Muscular projections of these cells extend in radially direction, i.e., from body wall to the center of the septum, at a right angle to the anterior–posterior body axis (Fig. 8A–C).

At the late metatrochophore stage, three more separate circular bands are added at the posterior end of the opisthosoma (*moc 4–6*, Figs. 3C, F, 5D–F). There is an outstripping development of circular muscle bands, which seem to outline the boundaries of the opisthosomal segments, followed by the development of muscle-free inner septal divisions (Figs. 2D, F–H, 5C, 6A, 8C).

## Competent larva

The length of the competent larva of *S. fiordicum* is up to 900–1000 um (Fig. 4A). The body consists of prostomium, peristomium, forepart (2nd segment), trunk (3rd segment), more than six opisthosomal and a pygidial lobe (Fig. 5G–I). In the competent larva, the prototroch almost disappears. In the peristomium, there is an anlage of tentacle and a dorsal ciliated spot. In the second segment (“forepart segment” in adult frenulates), the frenulum (*f*), or bridle, appears. The third segment (trunk segment of adult frenulates) is delineated anteriorly and posteriorly by septa (*s1*), and in the middle part, the chaetae of the annula, extend externally (Fig. 4A). The first segment of the opisthosoma, between the first septum and the mesotroch, is relatively long. In the relaxed larvae, this segment exceeds the length of all subsequent segments of the opisthosoma (Fig. 4A–E).

*Longitudinal musculature* Gradual formation of the continuous layer of longitudinal muscles continues. In the trunk segment (3rd segment), the longitudinal fibers of all four muscle strands run widely so that they almost form an almost continuous layer of longitudinal muscles (Fig. 4C, D). The longitudinal musculature assists burrowing: the competent larvae burrow into the sediment head downward, their rotating and bending posterior extremities assist digging into the sediment (Additional file 4).

*Circular musculature* Development of the circular muscles (*mc*) leads to the formation of the dense muscular corset in the anterior part of the larva (Figs. 4D, E, 5G–I). Contraction of the circular muscles facilitates the burrowing of the competent larva (Additional file 5).

On the dorsal side posterior to the prototroch, the bud of the unpaired tentacle (*tb*) appears, and a muscle ring forms at the base of the tentacular anlage (Figs. 4A–D, 5H, I).

In the competent larva, the trunk (3rd) segment lengthens and forms two parts (Fig. 4A). In the anterior part (before the annular chaetae), 4–5 circular bundles are visible (Fig. 4C), and in the posterior part of the body (behind the annular chaetae), there are no circular muscles at all (Fig. 4G).

*Septa and septal musculature*. At the stage of the competent larva, a distinct circular muscle band (*mf*) is visible in the middle of the anterior body segment (Fig. 4E). We assume this muscle band correlates with the position of the third septum (*sIII*) which divides the forepart + trunk tagma into two segments (forepart and trunk, or 2nd and 3rd segments) (Fig. 4A, C). These two segments (following the peristomium or 1^st^ segment) grow without further divisions into the adult stage. The anterior segment is termed the forepart (following Southward [46]), which is equivalent to the "fused protosome and mesosome”, according to the now abandoned deuterostomic concept of Ivanov [47]. The posterior segment is termed the trunk (following Southward [46]) and is equivalent to the "metasome", according to the now abandoned deuterostomic concept of Ivanov [47]). In the 2^nd^ body segment (forepart) there is a frenulum (*f*) (Fig. 4A–D). It is also known as the bridle, a characteristic structure found in all the currently described frenulates (see, for example, Ivanov [27]; Southward et al. [48]). In the 3rd segment (trunk) there are annular chaetae that delimit the preannular (genital) and postannular (trophosomal) parts (Fig. 4A, C, G).

The posterior circular bands and septa are formed one by one at the very end of the opisthosoma. We detected a seventh circular band in the competent larvae (Figs. 4H, 5G–I). Later in the post-larval stages a new circular band and a new septum form at the very posterior end of the juvenile. Adults of *S. fiordicum* have up to 21 muscle septa in the opisthosoma.

Within each circular opisthosomal muscle band, the number of muscle fibers increases with the age of the larva. For example, from 7 to 12 fibers within the bundle located behind the mesotroch (compare Figs. 2F, 4H) [27, 46–48].

*Musculature of chaetae* Although the chaetal musculature of the first segment of the opisthosoma is laid down already at the stage of the early metatrochophore (Figs. 2C, H), but the chaetae are actively used only by the competent larva. The larva can easily anchor the body in the sediment by the chaetae (Additional file 6).

**Fig. 10:**
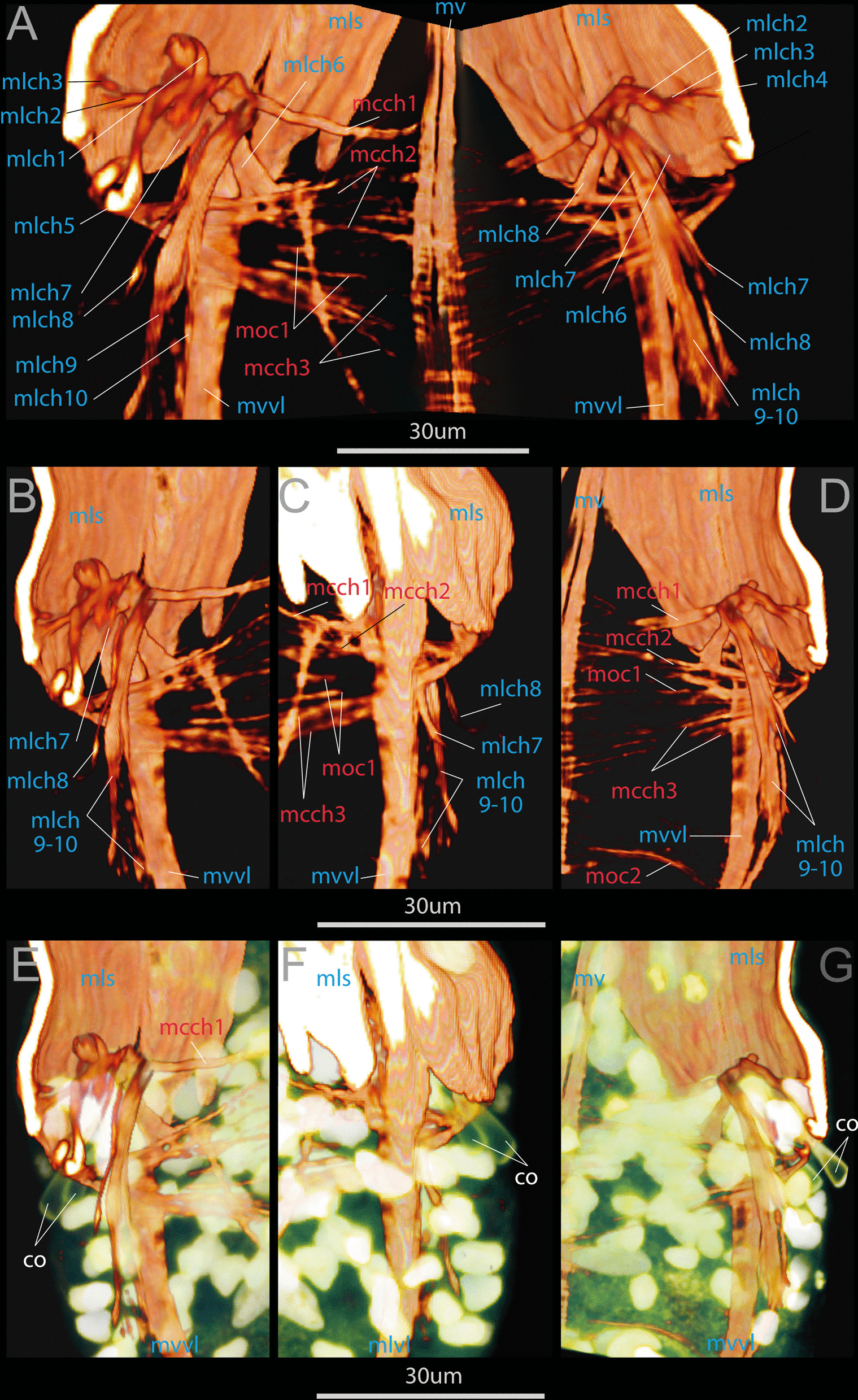
Muscles of the opisthosomal chaetae in the competent larva of *Siboglinum fiordicum*. Surface rendering in AMIRA of the longitudinal muscles of the first septum between trunk segment and the first segment of the opisthosoma**.** Musculature is shown in red; nuclei stained with DAPI as well as autofluoresce of the opisthosomal chaetae are shown in yellow. **A** Ventral view from the inside the larva body; **B**, **E** ventral view of the left side from the inside the larva body; **C**, **F** the same fragment from the outside; **D**, **G **ventral view of the right side from the inside. co—opisthosomal chaetae; mcch1-3—circular muscles of the chaetal apparatus, mlch1-10—longitudinal muscles of the chaetal apparatus, mls—longitudinal muscles of the first septum, mvvl—ventral component of the ventrolateral longitudinal muscle strand, moc1-2—the first and the second circular muscle bundles in the opisthosoma, mv—ventral longitudinal strand

**Fig. 11:**
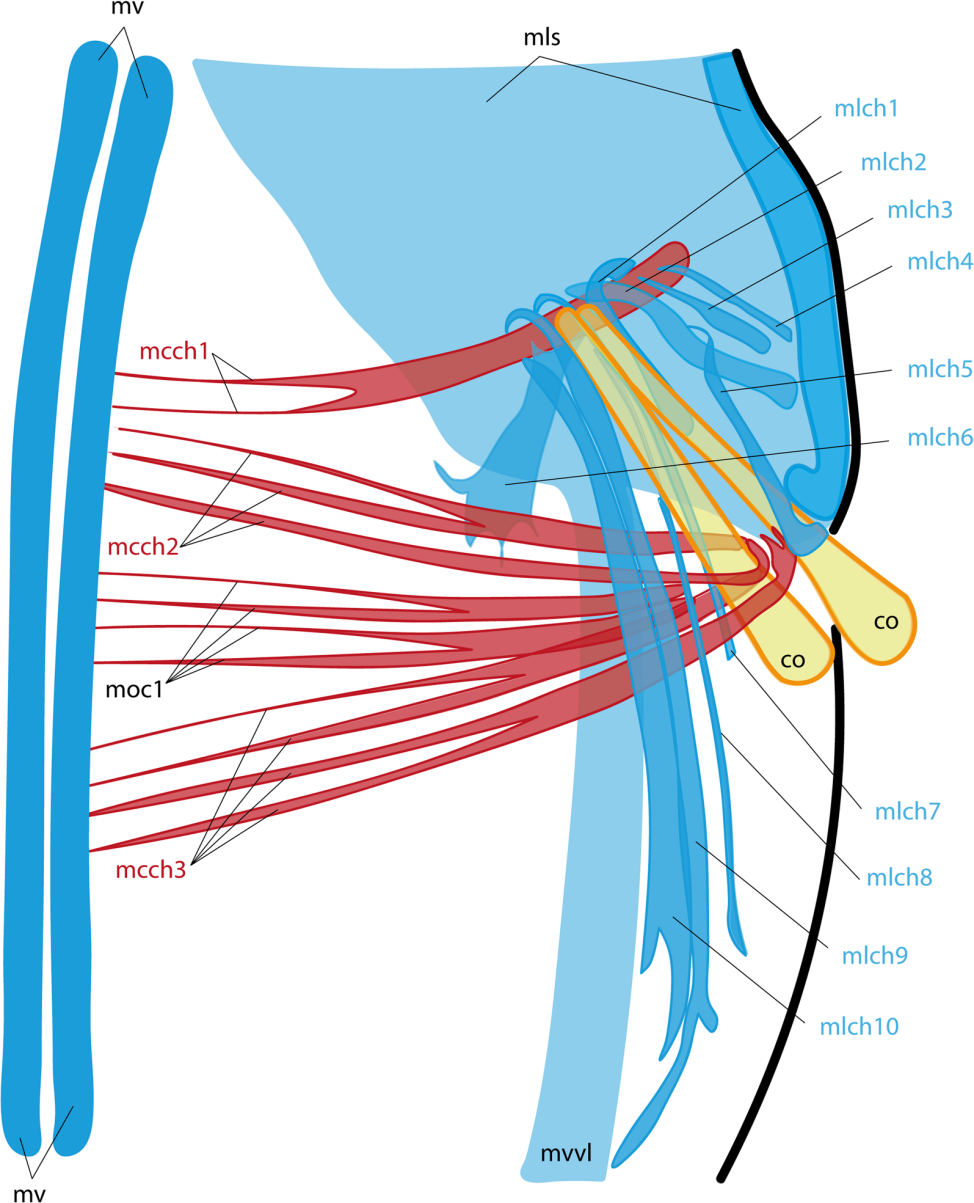
Schematic drawings of muscles of the opisthosomal chaetae in the competent larva of *Siboglinum fiordicum.* The scheme is based on the CLSM data. The ventral view of the level of the opisthosomal chaetae from the inside of the larva. Longitudinal muscles are shown in blue, circular muscles are in red, chaetae—in yellow. co—opisthosomal chaetae; mcch1-3—circular muscles of the chaetal apparatus, mlch1-10—longitudinal muscles of the chaetal apparatus, mls—longitudinal muscles of the first septum, mvvl—ventral component of the ventrolateral longitudinal muscle strand, moc1—the first circular muscle bundles in the opisthosoma, mv—ventral longitudinal strand

The movement of the opisthosomal chaetae is controlled by both specialized chaetal muscles and somatic muscle bundles. The latter include the longitudinal septal musculature (*mls*) and the circular septal musculature (*moc1*) (Figs. 10, 11). The chaetal muscles include separate circular and longitudinal muscle bundles. In total, there are 3 circular muscle bundles (*mcch1-3*) and about 10 longitudinal muscle bundles (*mlch1-10*) for each pair of chaetae (Figs. 2F–H, 10, 11). The circular muscles of the chaetal apparatus lie in the ventro-lateral and dorso-lateral directions (*mcch1-3*). Among the circular muscles, the first one (*mcch1*) stands out, one end reaching the ventral strand of the longitudinal muscles (mv), and the other attaching the body wall at the level of the septal muscles (*mls*) (Figs. 10A, 11). The first circular muscle lies at the base of the chaetae in such a way that the ends of the chaetae "abut against it". This circular muscle serves also as an anchor for almost all longitudinal muscles of the chaetae apparatus (*mlch1-4, 6-10*) (Fig. 10A–D). The rest of the circular chaetal muscles originate at the ventral longitudinal strand and supply the distal ends of the chaetae.

The longitudinal muscles of the chaetal apparatus attach with their distal end to the body wall either at the level of the first segment (*mlch1-6*) or at the level of the second segment (*mlch7-10*). The proximal end of the longitudinal chaetal muscles anchors either on the chaetae (*mlch5*) or on the first circular muscle or in the immediate vicinity of it (*mlch1-4, 6-10*) (Fig. 10A–D).

**Fig. 12:**
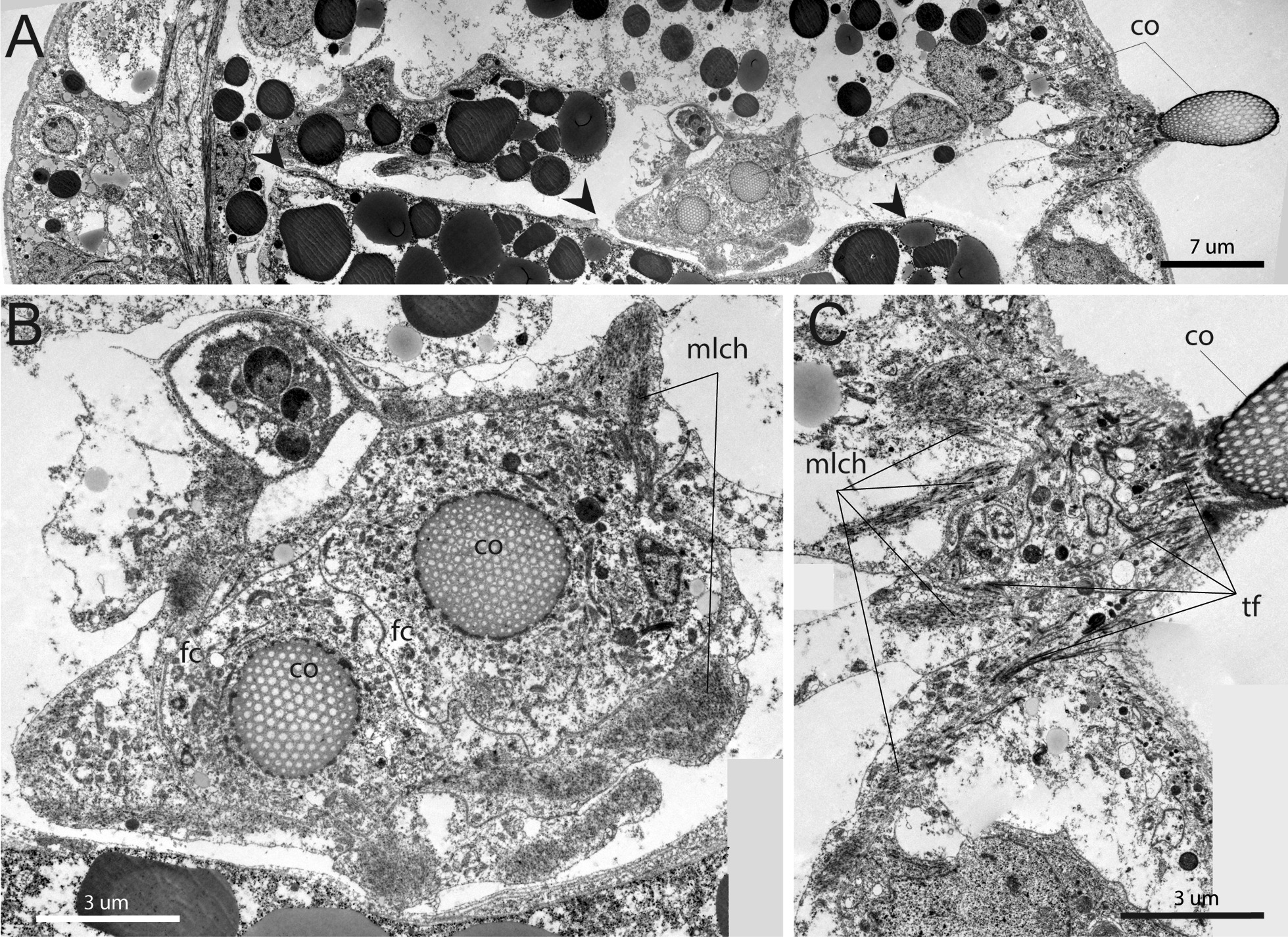
Ultrastructure of chaetal muscles of the late metatrochophore of *Siboglinum fiordicum*. **A–C** Ultrastructure of the muscle fibers of the chaetal sacs at the parasagittal sections. **A** Overview of the chaetae position in the first opisthosomal segment; arrowheads show the non-muscular second septum. **B** Close-up of the chaetal sac formed by the longitudinal muscle bundles and follicle cells chaetoblasts. **C** The chaetae raised by muscles, the muscles are attached to the cuticle through tonofilaments. fc—follicle cell, co—opisthosomal chaetae, mlch—longitudinal muscles of the chaetal apparatus, tf—tonofilament

The complicated musculature of the chaetal sacs is organized by the myoepithelial cells (Fig. 12A). All cells are connected via adherence junctions and have large muscular projections (Fig. 12B). Some of myoepithelial cells form the circular muscle lining, which envelops the follicle cells and chaetae (*co*) (Fig. 12B). Other cells form the longitudinal muscles of chaetal sac (Fig. 12C). These cells extend between the layers of extracellular matrix of the chaetal sac and the epithelium of the body wall. On both sides, the myofilaments adhere to the extracellular matrix via hemidesmosomes. Myofilaments are mostly passed in a longitudinal direction. Interestingly, myofilaments are attached peripherally to the extracellular matrix exactly where the thick bundles of electron-dense tonofilaments (*tf*) of the epithelial cells are attached on the opposite side of the ecm basal membrane. These tonofilaments extend into tips of microvilli and attach to the cuticle (Fig. 12C). Thus, the myofilaments of the longitudinal muscles of the chaetal sac indirectly attach to the cuticle via thick tonofilaments of the epidermal cells.

The annular chaetae (*ca*) at the stage of the competent larva are not yet fully formed; therefore, we assume that the musculature also is not fully formed. At the studied stages (starting with the early trochophore), we see that four pairs of annular chaetae are controlled by their own muscles, which includes a pair of circular bundles (*mca*) and longitudinal bundles that extend from the ventrolateral longitudinal muscle strands (*mvl*) (Figs. 2G, 3B, 4G).

## Discussion

We described the muscular architecture of the frenulate larvae *Siboglinum fiordicum* employing a combination of F-actin staining of muscle fibres and ultrastructural studies, in order to understand the larval body plan and its locomotory adaptations. In addition, we described the heterochronous formation of segments and defined their boundaries.

*Sequence of septa formation and regionalization of the body* In the frenulate larva the border between the prostomium and the peristomium, bearing the ventral mouth and dorsal tentacle, is distinguishable and the regionalization follows that of other annelid larvae [38, 49]**.** But the division of the rest of the body raised questions [33, 50].

According to the classical concept of annelid development, after the simultaneous formation of the three anterior larval segments, the next segments are sequentially added from the posterior growth zone [5, 51–53]. This is well studied in an errant nereid with the homonomous body plan, *Platynereis dumerili* [1, 2]. However, in *S. fiordicum* with a heteronomous body plan, we do not observe neither a simultaneous formation of the first three segments nor a distinct posterior-anterior sequence of segment formation. Here, the first septum formed instead cut off the posterior tagma, the opisthosoma. Then six segments are almost simultaneously laid within the opisthosomal tagma, whereafter the 2nd and 3rd segments are divided anteriorly. Then the rest of the opisthosomal segments are formed in the posterior end of the opisthosoma one by one. In sum*,* the first septum divides the body into two tagmata, and later the segmentation occurs within the tagmata. In a very similar way, the formation of segments in the heteronomous chaetopterid *Chaetopterus variopedatus* occurs within the body tagmata and in an altered order, not strictly from posterior to anterior [44]. The absence of the postero-anterior sequence of the anlagen of segments is also known in the heteronomous serpulid *Hydroides elegans* in the group Sedentaria [54]. Adult *H. elegans* has seven thoracic segments and 30–60 abdominal segments. In the larvae, three anterior thoracic segments are first formed, then the anterior four abdominal segments are formed, then four posterior thoracic segments are formed, after which the remaining abdominal segments are formed one by one in the posterior growth zone [55, 56]. This makes us speculate whether a posterior growth zone and a clear posterior-anterior growth pattern may not be the only way of segment formation in Annelida. It is likely that, annelids, with a sedentary-tubicolous lifestyle, need different parts of the body to have mobility (and, thus, be divided into segments) at certain stages of development associated with settlement and the transition to a sedentary mode of life. Therefore, these annelids have no clear postero-anterior growth pattern. For example, in *Siboglinum fiordicum*, its segmental formation is associated with the characteristics of the life cycle and ecological adaptations of the larvae. The first to appear are the opisthosomal segments at the posterior end of the larva, which is associated with the mobility of the posterior end of the larva's body necessary for emergence from the mother tube and for subsequent burial of the larva in the sediment [57].

Oweniids, which phylogenetically occupy a sister position in relation to all other annelids, also have a heteronomous body and a tubicolous lifestyle [11, 12, 58]. But the first 11–12 segments of a young worm of *Owenia fusiformis* which is formed inside the planktonic mitraria-larva, arise from the paired teloblastic cells in a posterior-anterior sequence [59]. Probably due to the "comfortable conditions" inside the mitraria larva, there is no need for any mobility of different parts of the worm's body. Therefore, the segments in heteronomous *Owenia* might form in the posterior-anterior sequence, and not in an altered chronology or sequence as in *Siboglinum, Chaetopterus* and *Hydroides* [4, 42, 44, 45, 60].

*Larval movement* The somatic muscles are responsible for the generalized movement of the larvae from early metatrochophores to competent larvae. The strands of the longitudinal muscles provide bending of the larva's body in various directions relative to the longitudinal axis (Additional files 2–4). The development of a longitudinal layer of musculature in the trunk and in the opisthosoma of the late metatrochophores and competent larvae indicates a special role of the posterior half of the larva's body in generating body movement, including burrowing into sediment. The circular musculature, as an antagonist of the longitudinal musculature, provides the elongation of the larva's body along the longitudinal axis. In the competent larvae, the significant development of a continuous layer of circular muscle in the anterior half of the body indicates that this end is intensely shrinking and stretching along the longitudinal axis, which is important for successful penetration into the sediment (Additional files 5). In the late metatrochophores and competent larvae, the muscular apparatus of the chaetae of the opisthosoma is completely formed, and used for movement and anchoring (Additional files 6).

In swimming early and late metatrochophores, there is a wide neurotroch, which presumably serves as a sense organ and employed for ciliary gliding, the ventro-lateral longitudinal muscle strand may facilitate “exploratory” or “search” side to side movements. The strands of the longitudinal muscles provide bending of the larva's body in various directions along the longitudinal axis. The positions and widths of the longitudinal muscle strands indicate that the larvae may bend mainly dorsally and ventrally due to the widest dorsal longitudinal strand and joint work of ventrolateral and ventral longitudinal strands. This undulatory body wave-movements might help them to crawl or burrow into sediment.

Particularly intensive muscle development is seen in the “preannular” area of the trunk segment (the trunk area which is anterior to the annular chaetae), which makes about ¼ of the total length of the early metatrochophore, and also in the longitudinal muscles of the first septum of the opisthosoma. In metatrochophores and competent larvae (Figs. 3, 4), the longitudinal muscles in the preannular area are so developed that they cover almost the entire perimeter except for narrow gaps. Most of this muscle "corset" is made up of the dorsal and ventrolateral strands, the ventral strand is still less developed. A solid layer of musculature in this area and the highly developed longitudinal muscles of the first septum of the opisthosoma may indicate that the posterior part of the body actively moves in the early and late metatrochophores, providing twisting of the posterior body and repulsion of the larva from the mother’s tube. Larvae can crawl towards the anterior end of the maternal tube or over the sediment by pushing the posterior end of the body in all directions. These muscles may be indirectly involved in the movement of the opisthosomal chaetae.

It seems to us that the unusual observed [dorso-ventral as well as left–right bending rather than left–right undulatory movements] twisting movements of the larva are reflected in the unusual organization of the longitudinal strands of the frenulates: there is one dorsal and one ventral and two ventrolateral strands of the longitudinal muscles, while the more popular position of the longitudinal bundles in annelids is pairs of dorsal and ventral ones [16, 61].

Circular muscles are likely more important for burrowing forms, but are not required for animals that move using parapodia or cilia [61]. This explains why the anterior end of the *Siboglinum* larvae is equipped with a continuous layer of circular muscles, presumably specialized to burrow into sediment using peristalsis [57] (Additional file 4). But a distinctive feature of the *Siboglinum* larvae is that the circular muscles are seemingly located within or internal to the longitudinal muscles (Fig. 6D). It is not yet clear what advantage this gives *Siboglinum* but transverse muscles lying internal to the longitudinal muscles have also been observed in members of phylogenetically widespread families such as Sphaerodoridae [62–65], Nerillidae [62], Magelonidae and Spionidae [66].

*Chaetal movement* Annular chaetae do not function in the studied larval stages; adult frenulates use them to attach to the tube. At the late larval stages, 4 pairs of chaetae of the first segment of the opisthosoma are very mobile (an additional movie file shows this in more detail, see Additional file 1), and their movement is carried out by a set of longitudinal and circular muscle bands (Figs. 10, 11). From the muscular reconstructions we suggest the following functionality of the various muscles: each pair is located in a single chaetal sac and the “neighbors” are controlled by the muscle bundles synchronously (Fig. 12). The first annular muscle (*mcch1*) serves as an anchor for almost all longitudinal bundles of the chaetal apparatus (except *mlch5*). The longitudinal bundles (*mlch1-10*) provide forward and backward movement of the chaetae, but their joint work with mcch1 provides twisting movements of the chaetae. Longitudinal bundles having an anterior position (*mlch1-4*) provide backward movement of the chaetae, the mlch5 bundle attached to the chaetae and mlch6 bundle move the chaetae dorsally and slightly forward. The longitudinal muscles, which have a posterior attachment to the body wall (*mlch7-10*), serve as levers to move the chaetae forward. Mlch7-8 move the chaetae forward and dorsally, while mlch9-10 move forward and ventrally. The circular muscles mcch2-3 are responsible for the retraction and descent of the chaetae, pressing them against the longitudinal axis of the body and decreasing the angle with the longitudinal axis.

## Conclusions

Our results show that the septum formation in the *Siboglinum fiordicum* did not follow a strict temporal anterior to posterior sequence described as the ground pattern in annelids. Instead, the first septum divides the body into two tagmata. Later the segments are laid down within these tagmata: first in the posterior opisthosoma tagma, later in anterior forepart-trunk tagma. We consider the larval development of *S. fiordicum* as heterochronous, in that segments in each of the body tagmata develop at different times. This heterochronous larval development has likely evolved together with the heteronomous adult body form (specialized body regions) found in *S. fiordicum*. Growth patterns lacking a strict anterior–posterior sequence of segment formation are also known from the heteronomous *Hydroides* and *Chaetopterus*. They contrast with classical studies of annelid development showing that segmented annelids usually generate their first (three) larval segments simultaneously while later segments are sequentially added from a posterior growth zone, e.g. in *P. dumerilii*.

We suggest that during the settlement of the larvae and transition to a sedentary lifestyle, heterochrony is manifested in development. After all, the adults of the heteronomous annelids *Siboglinum*, *Chaetopterus*, and *Hydroides*, with the altered sequence of the segments' formation, have a sedentary-tubicolous lifestyle. And at different stages of development of the larvae (i.e. a stage of searching for a suitable substrate, or a stage of burying/settling in the sediment), the mobility of a certain part of the body is required, which is conditioned by the appearance of segments in this body part.

## Methods

Specimens of *S. fiordicum* were collected at 18–35 m in the Ypsesund Strait (North Sea) in the close vicinity of the Espegrend Marine Biological Station, University of Bergen, Norway. Later in the lab, trochophores and metatrochophores were extracted from female tubes. Competent larvae were raised from metatrochophores in laboratory culture during 1–2 weeks. The rate of development is influenced by temperature. An approximate estimation of the rate is given by Bakke [57, 67, 68].

*Scanning electron microscopy (SEM)* Larvae were anesthetized with 7% MgCl2, were fixed in 2.5% glutaraldehyde in 0,1 M cacodylate buffer with 5% sucrose and later postfixed in 1% osmium for 1.5 h, dehydrated, dried out at the critical point and sputter coated with a platinum. SEM studies were performed on the JEOL JSM microscopes (JEOL Ltd., Tokyo, Japan) at the Laboratory of Electron Microscopy of Moscow State University.

*Confocal laser scanning microscopy (CLSM)* Larvae were anesthetized with 7% MgCl2, fixed with 4% paraformaldehyde over night at 4 °C and then rinsed six times in phosphate-buffered saline (PBS). After 1 h preincubation in PTA (PBS + 5%Triton, 0.05%NaN3 and 0.25% BSA) animals were stained for 1 h with Alexa Fluor 488-labeled phalloidin (INVITROGEN, Carlsbad, USA) dissolved in PTA, to visualize F-actin. For cilia visualization the monoclonal mouse acetylated α-tubulin (final concentration 1:400; Sigma-Aldrich, T6793) was used (with CY5 labeled secondary antibody directed against mouse; Jackson Immuno-Research, West Grove, PA, USA). Samples were preincubated for 1–2 h in PTA (PBS + 5% Triton-X, 0.05% NaN3, 0.25% bovine serum albumin, and 10% sucrose). Afterwards, samples were incubated for up to 48 h at RT in the primary antibody, both made with PTA (with 1% Triton). The samples were then thoroughly washed through several shifts with PTA over 6 h and then incubated overnight at RT in the two respective secondary antibodies conjugated with fluorochromes and mixed in PTA. Stained animals were mounted in Vectashield® Antifade Mounting Medium with DAPI (Vector Laboratories, Burlingame, CA, USA) and examined with Olympus Fluoview FV-1000 confocal laser scanning microscope (CLSM) in the University of Copenhagen. Z stacks of scans were projected into 2D-images and 3D reconstructions in Fiji and AMIRA 2020 (ThermoFischer Scientific) and used for shematic drawings made in Adobe Illustrator 2020. CLSM images were adjusted in Adobe Photoshop 2020 and assembled in Adobe Illustrator 2020.

*Ultrastructure studies* Specimens were fixed in 2.5% glutaraldehyde in 0.1 M cacodylate buffer with 5% sucrose and later postfixed in 1% osmium for 1.5 h. Prior to embedding in Spurr, specimens were dehydrated in alcohol series using standard protocol and thereafter polymerized for 20–24 h at 60 °C. The block was trimmed to the object and sectioned into semithin (500 nm) and ultrathin (30–40 nm) sections using a Leica EM UC7 ultramicrotome (LEICA MICROSYSTEMS, Wetzlar, Germany). Ultrathin sections were mounted on slot grids and mesh grids, contrasted with 1% uranyl acetate- and 4% lead citrate-solution. Transmission electron microscopy (TEM) performed with JEOL JEM-1011 equipped with digital camera ORIUS SC1000W, and JEOL JEM-1400 Flash equipped with Gatan Rio 9 fast CMOS 3k camera (JEOL Ltd., Tokyo, Japan) at the Laboratory of Electron Microscopy of Moscow State University. Later pictures were processed in Adobe Photoshop 2020 and assembled in Adobe Illustrator 2020.

*Videorecordings* To make a video, an iPhone 6S smartphone (Apple Inc.) and an iDu CamLab adapter (iDu Optics, USA) for a stereomicroscope on an iPhone are used. Video processing was done in Adobe Premiere Pro CS6 (Adobe Systems Incorporated, San-Jose, USA, 2012).

## Supplementary Information

**Additional file 1.** Typical ciliary swimming for trochophores of *Siboglinum fiordicum*. At this stage no muscle fibers are detected

**Additional file 2.** The early metatrochophores of *S. fiordicum* swim by beating of cilia as well as bend their bodies by contracting the longitudinal muscular strands

**Additional file 3.** Late metatrochophores of *S. fiordicum* stop actively swimming, they often lie at the bottom of the glass dish or sediment but continue to bend the body. The bending movement occurs at the level of the posterior trunk, where only longitudinal muscles are present. Their muscular projections bear numerous finger-like outgrowths (Fig. 7A) that allows larvae to elongate body easily

**Additional file 4.** Competent larvae of *S. fiordicum* burrow into the sediment by using the peristaltic contraction of the circular musculature of the forepart

**Additional file 5.** Competent larva of *S. fiordicum* burrow into the sediment, head downward, assisted by twisting motions of the posterior body

**Additional file 6.** Chaetae are often anchored in the sediment particles. Competent larvae of *S. fiordicum* control the chaetal movement by the now highly developed chaetal muscles

## Data Availability

All data (electron and confocal microphotographs, movies, and schemes) are kept by NRK and available by personal request.
